# Role and limitation of cell therapy in treating neurological diseases

**DOI:** 10.1002/ibra.12152

**Published:** 2024-03-12

**Authors:** Yu‐Qi Li, Peng‐Fei Li, Qian Tao, Issam J. A. Abuqeis, Yan‐Bin Xiyang

**Affiliations:** ^1^ State Key Laboratory of Primate Biomedical Research, Institute of Primate Translational Medicine Kunming University of Science and Technology Kunming China; ^2^ School of Basic Medicine Kunming Medical University Kunming China; ^3^ Department of Pharmacology and Toxicology, College of Pharmacology University of Arizona Tucson Arizona USA

**Keywords:** cell therapy, mechanism, neurological diseases, organoids

## Abstract

The central role of the brain in governing systemic functions within human physiology underscores its paramount significance as the focal point of physiological regulation. The brain, a highly sophisticated organ, orchestrates a diverse array of physiological processes encompassing motor control, sensory perception, cognition, emotion, and the regulation of vital functions, such as heartbeat, respiration, and hormonal equilibrium. A notable attribute of neurological diseases manifests as the depletion of neurons and the occurrence of tissue necrosis subsequent to injury. The transplantation of neural stem cells (NSCs) into the brain exhibits the potential for the replacement of lost neurons and the reconstruction of neural circuits. Furthermore, the transplantation of other types of cells in alternative locations can secrete nutritional factors that indirectly contribute to the restoration of nervous system equilibrium and the mitigation of neural inflammation. This review summarized a comprehensive investigation into the role of NSCs, hematopoietic stem cells, mesenchymal stem cells, and support cells like astrocytes and microglia in alleviating neurological deficits after cell infusion. Moreover, a thorough assessment was undertaken to discuss extant constraints in cellular transplantation therapies, concurrently delineating indispensable model‐based methodologies, specifically on organoids, which were essential for guiding prospective research initiatives in this specialized field.

## Graphical abstract

Different types of cell xenotransplantation for treating neuro‐related diseases. Mesenchymal stem cells, neural stem cells, microglia, astrocytes, and hematopoietic stem cells are used to treat various neurological‐related diseases, including A β accumulation, neuronal death, brain atrophy, traumatic brain injury, nerve fiber entanglement, and so forth.

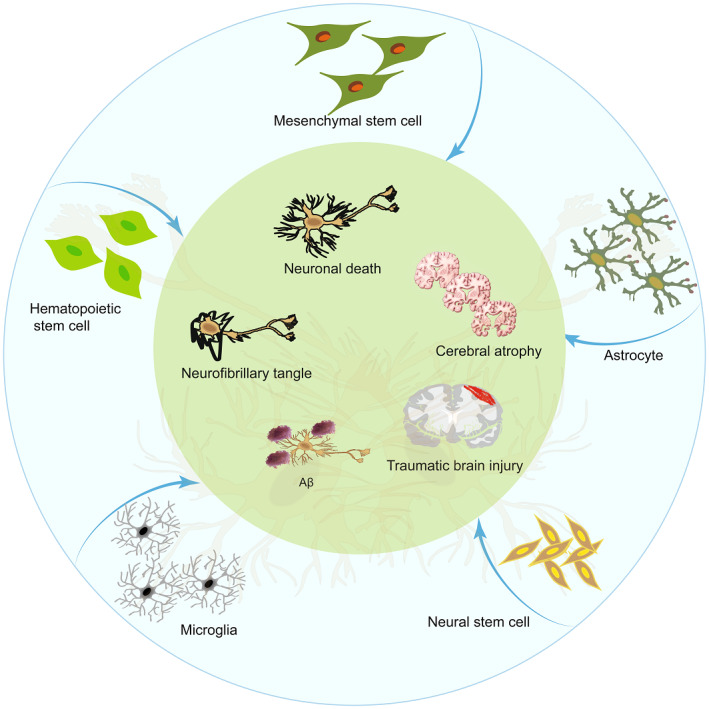

## 1 INTRODUCTION

The pathogenesis of neurological diseases is complex, involving multiple factors such as genetic mutations and the aging process.^1^, ^2^ Taking neurodegenerative diseases as an example, after the onset of the disease, significant characteristics include the loss of neurons, deficiencies in neurotrophic support, alterations in the neural microenvironment, localized tissue necrosis from injury, and increased gliosis.^3^, ^4^ If it can promote the regeneration of neurons and the reconstruction of neural circuits, it may promote the recovery of neurodegenerative diseases.^5^ Numerous studies have shown that cell therapy is effective for neurodegenerative diseases.^6^ As a consequence, the utilization of cell transplantation has emerged as an auspicious therapeutic modality.^7^ Neural stem cells (NSCs) possess the inherent capability to undergo differentiation into a spectrum of cell types, comprising astrocytes, oligodendrocytes, and neurons.^2^, ^8^ A wealth of research findings indicated that subsequent to NSC transplantation, a substantial degree of integration with host cells ensues, thereby facilitating the reconstruction of neural circuits and the restoration of motor and cognitive functions, as substantiated by observations in animal models.^5^, ^9^ Additionally, mesenchymal stem cells (MSCs) have strong immunomodulatory capabilities and secretion functions, allowing them to provide nourishment to locally injured tissues and effectively regulate the inflammatory microenvironment.^10^, ^11^ As the aging process unfolds, the manifestation of an inflammatory microenvironment assumes noteworthy prominence. The subtle intricacies of an immune microenvironment can instigate the activation of microglia, designating these cellular entities as a primary locus for addressing therapeutic interventions in the context of neurodegenerative diseases.^12^, ^13^ In conditions characterized by abnormal microglia activation, strategies aimed at suppressing this irregularity. Hematopoietic stem cells (HSCs) can migrate to the brain and develop into microglia, replacing host microglia through exogenous transplantation, and establishing transplanted HSCs within the brain to reconstruct its microenvironment are crucial in neurodegenerative disease therapeutics.^14^ Astrocytes are the most widely distributed type of cells in the brain, located between the cell body and its processes of nerve cells, supporting and guiding neurons, enhancing their survival, and promoting synaptic connections between neurons.^15^, ^16^, ^17^ Similarly, astrocytes play a multifaceted role in the brain, undertaking various functions, such as supplying nourishment, reinforcing neuronal structure, and facilitating the clearance of debris, making them potential to treat neurological diseases.^18^ Together, this review summarized the role of NSCs, MSCs, astrocytes, microglia, and HSCs in the treatment of neurological diseases through transplantation. Furthermore, this study conducts a critical assessment of the existing limitations inherent in current transplantation methods and delves into potential avenues like organoids for optimizing therapeutic strategies.

## 2 MECHANISMS OF HETEROGENEOUS CELL TRANSPLANTATION FOR NEUROLOGICAL DISEASE THERAPY

### 2.1 Transplantation of NSCs for neurological diseases

**Figure 1:**
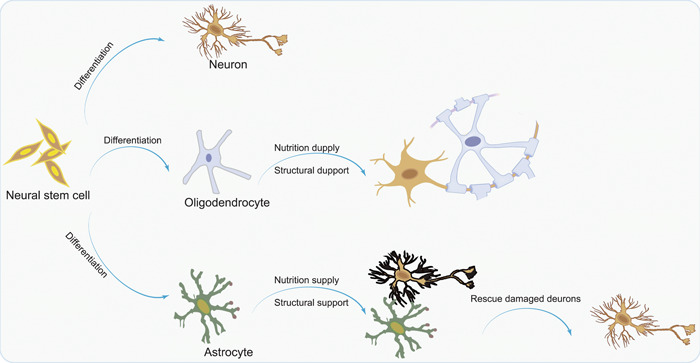
NSCs differentiate into neurons to supplement the neurons lost in the brain due to diseases, oligodendrocytes to encapsulate neurons and prevent abnormal neuronal discharge, and astrocytes, providing both nutrition and structural support for neurons. NSC, neural stem cell. [Color figure can be viewed at wileyonlinelibrary.com]

Neurodegenerative diseases, colloquially termed the “silent killers” within the geriatric demographic, predominantly involve the loss of neurons, a phenomenon further compounded by the heightened susceptibility inherent in mature nervous systems.^4^, ^19^ NSCs, residing within the central nervous system, possess the capacity to generate diverse neural components, encompassing neurons, astrocytes, and oligodendrocytes.^16^, ^20^ Predominantly localized in regions, such as the subventricular zone, dentate gyrus of the hippocampus, and the hypothalamus, NSCs assume a pivotal role in the therapeutic management of neurodegenerative diseases,^21^, ^22^, ^23^ and manifest the capability to undergo differentiation into a spectrum of cell types, including neurons, astrocytes, and oligodendrocytes.^24^ The consequential roles played by these differentiated cell types are crucial, encompassing the replacement of lost neurons, provision of structural support, and facilitation of the repair of damaged myelin^25^, ^26^ (Figure 1). Neurodegenerative and traumatic diseases affecting the nervous system, such as axonal malnutrition,^27^ Parkinson's disease,^28^ stroke,^29^ brain injury,^30^ and spinal cord injury,^31^, ^32^, ^33^ lead to tissue necrosis and physiological damage in brain tissue. However, the endogenous production of NSCs significantly diminishes as the nervous system matures, thereby restricting their potential to effectively repair neural damage caused by these conditions. Consequently, supplementing lost neurons or stimulating their regeneration typically necessitates an external source.

A multitude of studies underscore the profound therapeutic impacts of NSCs on various ailments, such as Parkinson's and Huntington's diseases. Transplanted NSCs not only assimilate into the recipient's brain, establishing novel synaptic connections but also secrete neurotrophic factors that modulate immune responses and confer neuroprotective properties.^34^, ^35^, ^36^ As a result, allogeneic cell transplantation has emerged as a viable approach to address physical brain injuries and physiological abnormalities. Studies have shown that NSCs can provide nourishment to damaged cells and regulate immune and metabolic pathways through a bystander effect.^37^, ^38^, ^39^ These NSCs are initially obtained from the telencephalon and diencephalon of human fetal brains after selective pregnancy terminations between 10 and 12 weeks postconception.^40^ Before transplantation into patients, the NSCs undergo in vitro expansion, quality control, and safety assessments at different dosage levels.^41^ The experimental findings furnish evidence that the transplantation of NSCs is associated with a reduction in overall brain and gray matter volume loss.^42^ Furthermore, the examination of untargeted proteomics and metabolomics in the cerebrospinal fluid of subjects subjected to NSC transplantation indicates that these cells operate as neuroprotective agents, functioning in nutrient provision and immune system regulation.^43^

### 2.2 MSC transplantation for neurological diseases

**Figure 2:**
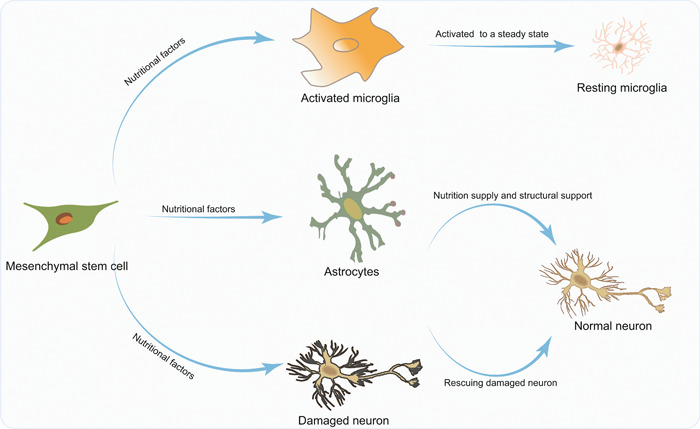
The mechanism involved in MSC transplantation for treating neurological diseases. MSCs release anti‐inflammatory factors to reduce abnormal activation of microglia and restore them to a resting state. They provide nutritional factors for astrocytes and neurons to reduce neuronal death. MSC, mesenchymal stem cell. [Color figure can be viewed at wileyonlinelibrary.com]

MSCs possess a remarkable capacity to differentiate into a wide array of cell types, including osteoblasts, chondrocytes, adipocytes, bone marrow stromal cells, and liver cells. Consequently, they hold significant promise in promoting neural regeneration following injury.^44^, ^45^ These MSCs can be obtained from various sources, such as umbilical cord blood, umbilical cords, amniotic membranes and fluid, bone marrow, and placenta, and are characterized by their limited immunogenicity. The extensive accessibility, expandability, minimal immunogenicity, and notable therapeutic efficacy of MSCs make them a prominent option for cell‐based therapies aimed at neurorelated disorders. MSCs possess a diverse range of capabilities, encompassing immunomodulation, angiogenesis, tissue remodeling, antiapoptosis, growth factor secretion, and nourishment (Figure 2).^46^, ^47^, ^48^ Collectively, these attributes facilitate the survival of host cells, tissue restoration, and the stimulation or differentiation of local progenitor cells.^49^

Additionally, the extracellular vesicles derived from MSCs during stroke exhibit a remarkable ability to selectively migrate toward sites of injury, thereby safeguarding neural tissues against additional necrotic expansion, promoting late‐stage vascularization, and releasing growth factors that facilitate tissue remodeling.^49^ In the context of cotransplantation with other cell types, MSCs exhibit the capability to stimulate the differentiation of NSCs and augment the survival rates of transplanted NSCs.^50^, ^51^ These distinctive attributes position MSCs as a promising therapeutic modality for mitigating inflammation and addressing neurodegenerative alterations linked to various neuro‐related diseases across diverse clinical scenarios.

### 2.3 Transplantation of astrocytes to treat neurological diseases

**Figure 3:**
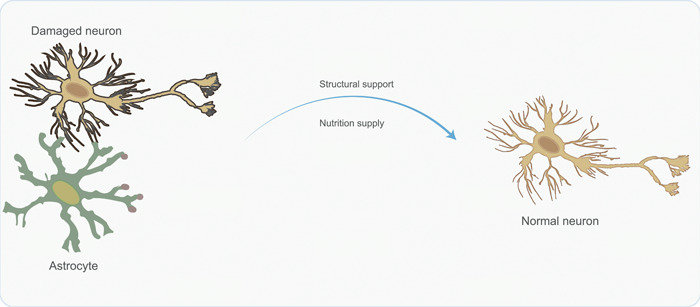
The mechanism involved in astrocyte transplantation for treating neurological diseases. Astrocytes provide nutrients and structural support to neurons and rescue them from death. [Color figure can be viewed at wileyonlinelibrary.com]

Astrocytes, derived from the neural epithelium, assume a crucial role in upholding homeostasis in the central nervous system.^15^, ^16^ They exhibit a wide array of functions, encompassing molecular to systemic domains, such as regulating ion equilibrium, providing metabolic support, modulating synaptic connections, safeguarding the blood–brain barrier, and clearing cellular debris^52^, ^53^ (Figure 3). Empirical evidence indicates that cotransplantation of NSCs and astrocytes yields substantial improvements in neural functionality in rodent models of ischemic stroke, surpassing the outcomes achieved through individual NSC transplantation.^54^, ^55^ Furthermore, this integrated approach notably mitigates the extent of brain infarction volume when contrasted with the sole transplantation of NSCs.^56^ Research findings indicate that the transplantation of mature neural glial progenitors in the adult brain facilitates the reorganization of the sensory cortex through their functional integration into the pre‐existing astrocyte–neuron circuitry.^57^ Furthermore, the introduction of glial precursor cells (GPCs) into the cortex of adult mice leads to the development of astrocytes with typical functional attributes following in vitro cultivation. These transplanted cells demonstrate remarkable abilities in terms of migration, differentiation, and long‐term integration, thereby preserving a more youthful state within the aging brain. Significantly, the transplantation of GPCs has demonstrated the ability to reverse age‐related sensory sensitivity.^58^ After spinal cord injury, astrocytes can promote the regeneration of damaged axons and the sprouting of undamaged axons by secreting appropriate extracellular matrix and neurotrophic factors, participating in immune regulation, and suppressing inflammatory responses. Studies have shown that transplantation of astrocyte lines can enhance axonal regeneration.^59^ The burgeoning body of empirical evidence emphasizes the potential efficacy of astrocyte transplantation as a promising therapeutic approach in the treatment of neurological disorders. This is particularly noteworthy for its impact on the modulation of neural circuitry and the rejuvenation of brain function.

### 2.4 The therapeutic potential of transplanted microglia in neurological diseases

Microglia, highly prevalent in cerebral tissue, are posited to have mesodermal origins and fulfill indispensable roles within the central nervous system.^12^ Their significant contributions encompass participation in brain development, maintenance of homeostasis, responsiveness to neural inflammation, and active engagement in aging processes.^60^, ^61^, ^62^ Particularly during early brain development, microglia actively participate in synaptic pruning, a vital process responsible for the elimination of excess synapses.^63^ However, in neurodegenerative disorders, such as advanced Alzheimer's disease, the aggregation of β‐amyloid proteins within the brain results in neurotoxic consequences, causing harm to neurons and triggering the activation of microglia.^64^, ^65^ Microglia demonstrate a range of functional phenotypes in response to various conditions. Following neural injury, they initially assume an activated M1 phenotype to facilitate the removal of cellular debris.^66^ Subsequently, they transition into an anti‐inflammatory M2 phenotype, releasing cytokines, such as interleukin‐4 (IL‐4) and IL‐10, thereby contributing to the mitigation of inflammation.^67^, ^68^

**Figure 4:**
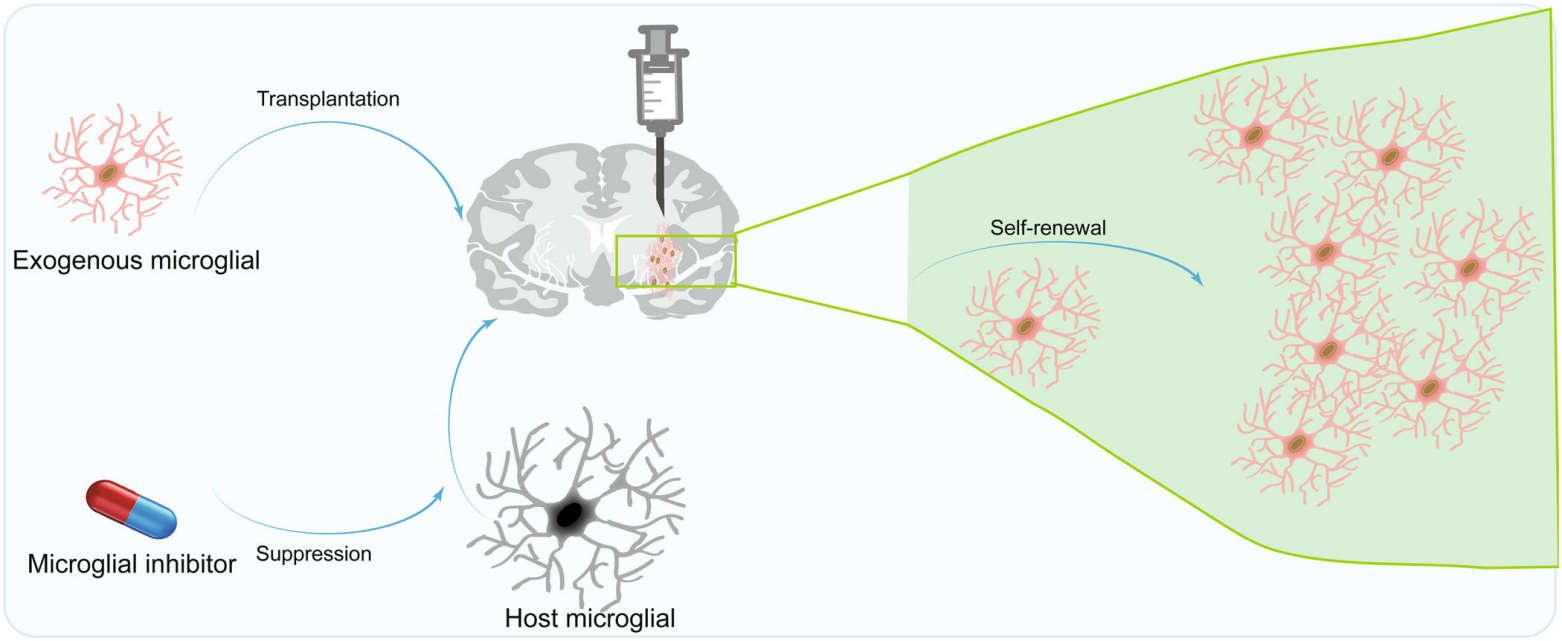
Mechanisms of transplanted microglia in addressing neurological diseases. The drug inhibits abnormal activation of microglia in the host's brain, while brain transplantation supplements healthy microglia. Microglia maintain brain homeostasis after self‐renewal. [Color figure can be viewed at wileyonlinelibrary.com]

Owing to their functions in surveillance and clearance processes, microglia have become a focal point in scientific investigations aimed at addressing related pathological conditions.^69^ Research demonstrated that the inhibition of microglia triggers their swift repopulation within the brain, resulting in the emergence of neuroprotective microglia^70^, ^71^, ^72^ (Figure 4). The efficacy of these regenerated microglia is contingent upon the signaling of IL‐6 via the soluble IL‐6 receptor, which facilitates adult neurogenesis and augments the viability of nascent neurons essential for cognitive processes.^73^ Significant advancements in the healing process and regeneration of axons within the spinal cord, a vital constituent of the central nervous system, have been documented through therapeutic interventions encompassing the transplantation of neonatal microglia or adult microglia treated with peptidase inhibitors.^12^, ^74^, ^75^ Nevertheless, the seamless integration of externally transplanted microglia into the recipient's brain continues to present a formidable obstacle. In 2020, three discrete techniques have been devised for the transplantation of microglia, each demonstrating efficacy in successfully engrafting the transplanted cells into the brain.^76^

### 2.5 HSC transplantation in neurological disease treatment

**Figure 5:**
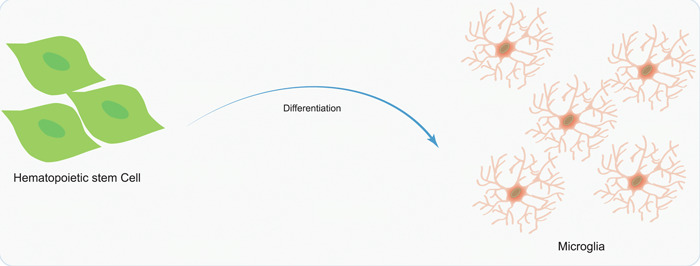
HSCs transplantation in neurological disease treatment. After HSCs transplantation, differentiation into microglia can migrate and colonize the brain to maintain neural homeostasis. HSC, hematopoietic stem cell. [Color figure can be viewed at wileyonlinelibrary.com]

HSCs are a cohort of undifferentiated cells located within hematopoietic tissues, responsible for generating all blood and immune cells.^77^, ^78^ These cells possess the distinctive ability to undergo specialization into fully developed red blood cells, white blood cells, and platelets, while simultaneously preserving the vital functions of self‐renewal and replication, which are imperative for regular hematopoiesis and immune response.^79^, ^80^ Recent investigations suggest that subsequent to transplantation, HSCs can differentiate into microglial cells and establish a presence within the cerebral region^81^, ^82^ (Figure 5). Research findings indicate that transplants obtained from HSCs and progenitor cells effectively mitigate various symptoms and manifestations in murine models of Alzheimer's disease.^14^, ^83^ In contrast to other mice affected by Alzheimer's, those who receive HSC transplants exhibit maintained memory and cognitive abilities, diminished neural inflammation, and a noteworthy reduction in the accumulation of β‐amyloid protein.^84^ Significantly, the research conducted by Marius Wernig and his team revealed that the systemic transplantation of HSCs has the ability to replace mutated microglia in triggering receptor expressed on myeloid cells 2‐mutant mice models of Alzheimer's disease, effectively restoring their functionality. These findings provide compelling evidence that HSC transplantation exhibits substantial potential as a prospective therapeutic strategy for forthcoming treatments of Alzheimer's disease.^14^

## 3 LIMITING FACTORS IN HETEROGENEOUS CELL TRANSPLANTATION FOR TREATING NEUROLOGICAL DISEASES

### 3.1 Immune rejection after cell transplantation

The field of organ and cell transplantation has made significant progress in the treatment of diverse medical conditions.^85^, ^86^, ^87^ Nevertheless, the pervasive challenge of immune rejection remains a significant impediment across all transplant procedures, ultimately contributing to instances of transplant failures.^88^, ^89^, ^90^ Currently, the management of immune rejection heavily relies on the administration of immunosuppressants, including cyclosporine, tacrolimus, and dexamethasone, with the primary objective of preventing rejection.^91^, ^92^, ^93^ However, prolonged usage of these medications frequently compromises the recipient's immune system, leading to infections and inadequate suppression of transplanted cells.^94^, ^95^, ^96^ According to research findings, regulatory T cells have been found to exert a temporary inhibitory effect on the proliferation of CD8+ T cells, thereby providing a limited defense against rejection for a duration of approximately 2 months.^97^ However, the unresolved matter of persistent immune rejection continues to be a matter of great concern. Specifically, in the context of intracranial cell transplantation for neurological disorders, the principal factor contributing to cell demise at the transplantation site is the substantial infiltration of T cells, B cells, and natural killer cells originating from blood vessels and peripheral tissues, which subsequently triggers immune rejection and self‐inflammation.^98^, ^99^ Dendritic cells (DCs) are the most functional specialized antigen‐presenting cells in the body.^100^, ^101^ In addition to playing an important role in the body's innate immune response, they are also responsible for initiating adaptive immune responses and influencing the types of immune responses, assisting in the development of immune effects.^102^ The surface of the DC membrane contains abundant major histocompatibility complex (MHC) class I and II, costimulatory molecules (CD80/B7‐1, CD86/B7‐2, CD40, CD40 ligands, etc.), intercellular adhesion molecule‐1 (ICAM‐1), ICAM‐2, ICAM‐3, and lymphocyte function related molecule‐1, which can stimulate initial T‐cell activation and proliferation.^103^, ^104^ The reactivation of microglia through infiltration of peripheral immune cells results in an upregulation of MHC class II, resembling that of peripheral DCs.^105^ This increased expression facilitates the presentation of antigen signals to infiltrating T cells, thereby establishing a connection between the immune systems of the peripheral and central nervous systems.^106^ This phenomenon persists until the restoration of the blood‐brain barrier, ultimately leading to the demise of transplanted cells originating from an external source.^107^, ^108^

### 3.2 Divergent brain development across species

Early‐stage transplantation experiments involving human‐origin cells are restricted to rodent and primate models as recipients, primarily due to ethical and moral considerations.^109^, ^110^ The existence of developmental disparities among primate, rodent, and human brains poses significant obstacles in establishing long‐lasting neural connections during interspecies cell transplantation.^111^, ^112^, ^113^ Notably, the brain stands out as a prominent differentiating factor between humans and animals.^114^, ^115^, ^116^ The human brain, which is approximately three times larger than that of chimpanzees, accommodates a greater number of neurons and glial cells, leading to an augmentation in neural pathways and the emergence of specialized functional regions, specifically associated with distinct human cognitive capabilities.^117^, ^118^, ^119^ The evolutionary mechanisms have exerted an influence on the initial formation of neurons in both chimpanzees and our human predecessors. In comparison to humans, the chimpanzee genome exhibits a higher abundance of Line 1 (L1) elements that are specific to the species, suggesting a heightened rate of L1 migration observed in non‐human primates beyond cultured induced pluripotent stem cells (iPSCs).^120^ The alterations in brain gene transcription over temporal and spatial dimensions exert an impact on the molecular, morphological, and functional characteristics of the brain. A study undertaken by the research team, led by J. Gray Camp encompasses the utilization of single‐cell transcriptomics and chromatin studies, spanning from early pluripotent cells such as embryonic stem cells and iPSCs to organoid structures, across various primate species, including humans, chimpanzees, and monkeys.^121^ Hence, a significant challenge in investigating the transplantation of human‐origin cells into different species lies in the incongruent developmental stages observed among these species.

## 4 FUTURE STRATEGIES FOR TREATING NEUROLOGICAL DISEASES

### 4.1 Brain organoid substitutes for animal models in neurological disease

Organoids are three‐dimensional structures that are created in a laboratory setting using pluripotent stem cells (PSCs) or adult stem cells.^122^, ^123^ These structures have the ability to self‐organize and mimic the cell compositions, tissue architectures, and functionalities of specific organs.^124^ The development of organoids was initially driven by advancements in human PSC technologies.^125^ Moscona,^126^ accomplished a noteworthy breakthrough in this domain and demonstrated the formation of organoid‐like structures through dissociation–aggregation experiments on embryonic neural tissue, resembling their in vivo counterparts. Zhang et al.,^127^ developed methods to direct the differentiation of human PSCs toward neural epithelial cells, enabling the creation of basic two‐dimensional models that mimic different cortical structures in the human brain. However, the crucial advancement toward the creation of the first human brain organoid occurred with the transition to three‐dimensional models. Nakano et al.,^128^ spearheaded this shift by cultivating PSCs‐derived neural epithelial cells in a fully three‐dimensional setting, guiding their maturation into optic nerve discs. This milestone played a pivotal role in establishing the foundation for further progress in retinal organoid development. Neurodegenerative diseases, such as Alzheimer's disease and Parkinson's disease, are primarily sporadic and intricate in their etiology.^129^, ^130^ The complex biological underpinnings of conditions like depression, autism spectrum disorders, and schizophrenia present challenges in replicating them in rodent models and assessing treatment effectiveness. Generally, non‐human primate models, which closely resemble human behaviors, are limited by high cost and rare sample availability for high‐throughput screenings. However, brain organoids cultivated in vitro offer a cost‐effective and efficient means to investigate the intricate mechanisms underlying these diseases.

### 4.2 Transplantation of in vitro‐cultured brain‐organoid tissues for neurological diseases

Understanding the development and dysfunction of the human brain is a major goal of neurobiology. The brain organoids created by human PSCs represent a promising method for brain repair.^131^ They acquire many structural features of the brain and increase the likelihood of patient matching and repair.^132^ A recent notable advancement has been made by scientists who successfully transplanted brain tissue organoids derived from human cells into mice suffering from stroke.^133^ This transplantation procedure effectively restored impaired tissue and reinstated motor functions.^134^, ^135^ Furthermore, upon transplantation into the visual cortex of rat brains, the brain organoids exhibited seamless integration into the host tissue, exhibiting progressive development of blood vessels, enlargement in size, generation of neuronal projections, and establishment of synaptic connections.^136^, ^137^ Moreover, the introduction of human brain organoids into the brains of neonatal rats, specifically those aged 2–3 days, a critical period characterized by ongoing neural connectivity development, resulted in the subsequent growth and occupation of approximately one‐third of the rat brain hemisphere by these transplanted human brain organoids.^132^ Notably, the neurons within these organoids successfully established functional connections with the existing neural circuitry, leading to the activation of these transplanted neural components and subsequent restoration of specific functionalities within the corresponding brain regions.^138^

## 5 CONCLUSIONS AND PROSPECTS

The brain, constituting a sophisticated system, integrates distinct cell populations to collectively execute multifaceted functions. Pathological conditions within the brain encompass a spectrum of underlying mechanisms, including neuronal malnutrition, extensive neuronal death, gene mutations, and glial cell activation.^139^ These mechanisms necessitate the exploration and development of diverse cell transplantation techniques. Cell transplantation, including astrocytes, microglia, and NSCs, has the potential to mitigate neuronal malnutrition, holding promise in ameliorating the inflammatory milieu within the brain.^17^ In addition, HSC transplantation facilitates the reconstruction of the hematopoietic system, wherein certain stem cells undergo differentiation into oligodendrocytes within the cerebral region.^14^ The transplantation of MSCs has been shown to mitigate brain inflammation by promoting neuronal growth and reinstating the brain's homeostasis via nutrient secretion.^140^ Nevertheless, the principal constraint of cell transplantation presently resides in immune suppression, thereby demanding the investigation of innovative immune‐suppressive approaches, combinations of diverse immune‐suppressive agents, and the minimization of their utilization.^141^, ^142^

As a result of ethical considerations, cells cultivated in vitro are limited to initial validation in animal models.^143^ Non‐human primates, which demonstrate phenotypes more closely resembling those of humans, are favored in disease animal model investigations; however, they encounter obstacles associated with costs. Brain organoids provide a practical avenue for the examination of genetic mutations or diseases associated with developmental anomalies.^144^ Serving as valuable research models, these organoids can be successfully engrafted into the host brain through transplantation.^132^ Nonetheless, it is imperative to acknowledge that brain organoids are subject to specific limitations arising from their enlarged size and volume, rendering them more applicable to scenarios involving surface damage in the cerebral cortex and comparable circumstances.

## AUTHOR CONTRIBUTIONS

Yu‐Qi Li wrote the original manuscript and drew the figures. Qian Tao collected literature. Peng‐Fei Li, Issam J. A. Abuqeis, and Yan‐Bin Xiyang fine‐tuned the whole article. Yan‐Bin Xiyang finalized and approved this paper. All authors have read and approved the final submitted manuscript.

## CONFLICT OF INTEREST STATEMENT

The authors declare no conflicts of interest.

## ETHICS STATEMENT

Not applicable.

## Data Availability

Not applicable.
